# Selective precipitation reaction: a novel diagnostic test for tissue pathology in Atlantic salmon, *Salmo salar*, infected with salmonid alphavirus (SAV3)

**DOI:** 10.1111/jfd.12587

**Published:** 2016-11-30

**Authors:** M Braceland, J Tinsley, D Cockerill, R Bickerdike, M F McLoughlin, P D Eckersall

**Affiliations:** ^1^ Institute of Institute of Biodiversity, Animal Health and Comparative Medicine University of Glasgow Glasgow UK; ^2^ BioMar Ltd. Grangemouth UK; ^3^ Marine Harvest Scotland Fort William UK; ^4^ Fish Vet Group Inverness UK; ^5^ Present address: Center for Aquaculture Technologies 20 Hope Street Souris PE Canada

**Keywords:** blood, diagnostics, pathology, protein precipitation, salmon, virus infection

## Abstract

While investigating biomarkers for infection with salmonid alphavirus (SAV), the cause of pancreas disease (PD), a selective precipitation reaction (SPR) has been discovered in serum which could be an on‐farm qualitative test and an in‐laboratory quantitative assay for health assessments in aquaculture. Mixing serum from Atlantic salmon, *Salmo salar*, with SAV infection with a sodium acetate buffer caused a visible precipitation which does not occur with serum from healthy salmon. Proteomic examination of the precipitate has revealed that the components are a mix of muscle proteins, for example enolase and aldolase, along with serum protein such as serotransferrin and complement C9. The assay has been optimized for molarity, pH, temperature and wavelength so that the precipitation can be measured as the change in optical density at 340 nm (Δ_340_). Application of the SPR assay to serum samples from a cohabitation trial of SAV infection in salmon showed that the Δ_340_ in infected fish rose from undetectable to a maximum at 6 weeks post‐infection correlating with histopathological score of pancreas, heart and muscle damage. This test may have a valuable role to play in the diagnostic evaluation of stock health in salmon.

## Introduction

Aquaculture is the world's fastest growing livestock producing industry with an average annual growth rate of 3.2 per cent being described by the Food and Agriculture Organisation from 1961 to 2009 (FAO, 2012) with an estimated 154 million tonnes of fish being produced in 2011, overtaking production of wild catch fisheries. One of the most economically important fin fish is Atlantic salmon*, Salmo salar*. Aquaculture of this species alone has increased from 1990 to 2010 at an average annual rate exceeding 9.2% (FAO, 2012); this growth is anticipated to continue to rise on the future. Despite this rise, the salmon farming industry is currently facing a number of barriers which may in the foreseeable future limit growth. One of the biggest challenges salmon aquaculture faces is that of infectious disease (Kibenge *et al*. 2012). Among these salmonid alphavirus (SAV) infection is a major problem causing salmon pancreas disease (PD) which has pathological effects on the pancreas, heart and skeletal muscles leading to morbidity and mortality of fish (McLoughlin & Graham 2007).

Minimizing the effects of such diseases is multifactorial relying on both effective prevention and treatment methodologies. To maximize and quantify the efficacy of a treatment, routine proactive health monitoring should be put in place within aquaculture. While diagnostic tools in the field have increased greatly in number and use in recent times (Adams & Thompson 2011), there is still a lack of both rapid on‐site and laboratory tests. Diagnosis of clinical disease relies heavily upon histopathology which is costly, time‐consuming, and requires destructive sampling thus limiting the proportion of a population which can feasibly be represented. Non‐destructive‐based technologies are centred on identifying pathogens on fish farm sites, although there is an increasing issue with presence of pathogens which may result in no clinical disease.

Pathological damage to tissues through necrosis or inflammatory responses during diseases such as PD is not only a welfare issue in aquaculture, but has significant economic consequences (Lerfall *et al*. 2012). While specific disease‐related mortality can be enumerated based on market value, the wider costs associated such as reduced feed utilization, an impaired immune system leading to susceptibility to secondary infections by other pathogens or parasite challenge, and increased downgrading of fillets at processing all contribute to a significant increase in the cost of farmed fish production (McLoughlin *et al*. 2002; Larsson *et al*. 2012). In the light of this, there is a growing interest in identifying biomolecules which may detect pathological damage and infection using non‐destructive techniques, such as analysis of plasma or serum after separation of blood samples.

Biomarkers of disease are any analytes that can distinguish between healthy and disease and that can be measured in samples of tissue or biological fluid (Mayeux 2004). While investigating serum biomarkers of infection in salmon with SAV, a discovery was made that a selective precipitation reaction (SPR) occurred when serum from infected fish was introduced to an appropriate buffer which did not occur when healthy sera were similarly introduced. The objective of this investigation was to characterize this discovery, in order to optimize and validate the applications of the SPR assay as a health assessment tool for salmon. Validation of the SPR assay was carried out on samples from clinically healthy Atlantic salmon and from experimentally infected fish which were from the SAV cohabitation challenge trial.

## Materials and methods

### SAV cohabitation challenge and histopathology

Sera from salmon with PD were obtained from the PD study described previously (Braceland *et al*. 2013, 2015). Briefly, 120 fish of 30 g (at the start of the challenge study) were maintained in each of 12 replicate tanks for a cohabitation challenge with SAV subtype 3. Trojan fish were infected with SAV 3 through intraperitoneal injection of SAV3 infected Chinook salmon embryo (CHSE) cell culture supernatant at ca. 10^5^ tissue culture infective dose (TCID)/fish and then introduced to cohabitant naïve fish. Nine cohabitant fish per tank were killed using a lethal overdose of anaesthetic (MS‐222, Pharmaq, UK) at 0, 2, 3, 4, 5, 6, 8, 10 and 12 weeks post‐challenge (Wpc) with W0pc fish removed prior to introduction of infected fish. Blood was collected from all of these fish (108 per time point) in non‐heparinized vacutainers, maintained overnight at 4 °C before centrifugation at 5000 ***g*** for 10 min, serum aspirated and stored at −20 °C prior to use in the SPR test. A random selection was made so that 54 samples per time point were subjected to the SPR test. In addition, pyloric caecae and pancreatic tissue (herein referred to in combination as pancreas), skeletal muscle and heart tissue were sampled from six (per tank) of these fish. Tissue was collected from standardized locations for histological assessment of virus‐induced pathology. Tissues were immediately fixed in 3.5% v/v formaldehyde in phosphate‐buffered saline pH 7.0 prior to paraffin wax processing, sectioned and stained with haematoxylin and eosin (H & E). Histological assessment of red and white muscle, pancreas and heart was carried out using a semi‐quantitative scoring system as first described by McLoughlin (2006) The experimental procedure carried out at VESO Vikan, Namsos, Norway and was approved by the Norwegian National Animal Research Authority (NARA) prior to the trial commencing (Braceland *et al*. 2013).

### Samples from aquaculture production sites

Serum, after separation from blood, and skeletal muscle lysates for use in optimization studies were obtained from healthy adult salmon (*n* = 37 for serum, *n* = 7 for muscle lysate) of an average weight of 3 kg and were prepared as described previously (Braceland *et al*. 2015). In brief, clinically healthy fish collected from an aquaculture production site (Ardnish, Scotland, UK) were treated with a lethal dose of anaesthetic (MS‐222, Pharmaq). Blood was taken in non‐heparinized vacutainers, maintained overnight at 4 °C before centrifugation at 10 000 × ***g*** for 15 min, after which serum was collected and stored at −80 °C. A pool of the sera, pool A (PLA) was prepared from 12 individuals, representing healthy salmon serum. Skeletal muscle samples were removed and frozen in dry ice and then stored until use at −80 °C. Tissues were grounded into a powder with a mortar and pestle and kept frozen by the periodic addition of liquid nitrogen. A ratio of 1 g tissue powder to 10 mL of buffer (20 mm Tris‐HCL, pH 7.5) was used to extract protein from the tissues. The resulting extract was transferred into a 15‐mL universal tube and centrifuged at 10 000 × ***g*** at 4 °C for 10 min, the supernatant removed and centrifugation repeated. The solid residue was discarded after each centrifugation. Finally, the tissue extract supernatant was passed through a 0.45‐μm syringe‐driven filter (Millipore, UK) and stored at −80 °C.

To determine a reference interval for the SPR test, further serum samples from 365 clinically healthy salmon of multiple weights, ages and sex were used using the method based on mean plus minus two standard deviations. Reference intervals for clinical biochemistry analytes for these samples were described previously (Braceland *et al*. 2016) and were from two further sites in the west of Scotland (UK). The samples were from fish of mixed sex but varied in age with one containing fish around 18 months old and the other containing fish around 24 months of age and with an average weight of 3.5 kg.

### Optimized SPR conditions for a quantitative laboratory test

The optimized buffer for SPR was 0.6 m sodium acetate (SA) at pH 5.6 prepared according to published buffer tables in Dawson *et al*. (1969). The optimization procedure for the quantitative SPR assay was based on a series of investigations where the assay parameters were varied using a microtitre plate spectrophotometer (FLUOstar OPTIMA, BMG Labtech). Individual optimization studies for the quantitative laboratory assay were undertaken to determine the effects of sample and buffer volume, absorbance wavelength, temperature, pH and molarity of sodium acetate (SA) buffer, and alternative buffers. Optimization was performed on pooled sera from clinically healthy salmon (PLA) and from fish from the SAV infection study at a time point which corresponds to known muscle pathology (W4pc pool). Skeletal muscle tissue lysate was also utilized to conserve limited stocks of serum from salmon with PD.

Buffer molarity and pH were optimized to limit the precipitation of normal circulatory proteins from serum, which would reduce sensitivity and specificity of the assay, while maximizing the SPR in serum from salmon with PD. Buffer molarity was examined using 0.05 m to 2 m SA buffer at pH 5.6, and buffer pH was investigated with 2 m SA buffer at pH from 3.7 to 5.6 using acetic acid to adjust pH. In these optimization studies, 15 μL serum or tissue extract was mixed in 245 μL buffer and absorbance was recorded as optical density (OD) at 340 nm over 60 min at 37 °C. The change in absorbance recorded as delta OD (ODΔ340 nm) was calculated by subtracting the initial reading (0 min) from the end point (60 min).

Optimization of assay conditions was also carried out for absorbance wavelength, temperature, sample to buffer ratio and with other buffers. A range of fixed wavelength detection filters were used to establish the optimal wavelength for observing precipitate formation with the microtitre plate reader. Wavelengths between 160 and 595 nm were assessed for the ability to yield absorbance maximum. Incubation temperature between 20 and 45 °C during the SPR assay was evaluated for the development of the reaction over 60 min. Optimizing sample volume was important to determine the amount of serum required from each individual animal and to minimize the amount of serum required to obtain a detectable reaction. Pooled diseased serum samples from 4 weeks post‐PD challenge (W4pc) or muscle lysate samples from 10 μl to 60 μl were used to detect a change in turbidity while minimizing sample volume. Sodium citrate, potassium acetate or ammonium sulphate (0.6 m at pH 5.6) were assessed for use in the quantitative assay to determine suitability of buffers.

To determine assay precision and intra‐assay coefficient of variance (CV), pools of sera from healthy or diseased fish from the SAV infection challenge were included as control in the optimized assay as low and high quality controls (QC), respectively.

### Optimized SPR conditions for a qualitative on‐farm test

Buffer conditions and sample volumes were identical to that of the quantitative test so that for the qualitative test, 15 μL of sample was added to 245 μL of 0.6 m SA buffer at pH 5.6 in a clear‐sided cuvette (Horiba). The solution was mixed, and the reaction observed over 60 min at room temperature. A positive result was confirmed when an opaque, white precipitate in the mixture was observed after 60 min. Further development of the reaction was assessed after 3 days incubation at room temperature.

### Two‐dimensional electrophoresis and mass spectrometry

To investigate the composition of the proteins forming the precipitate, separate pools of clinically healthy (W0pc) and diseased serum (W4pc) were prepared with equal volumes from each of these time points. Samples of the serum pools were added to 0.6 m SA pH 5.6 buffer in a ratio of 1:5 (serum:buffer) based on the evaluation of the effect of sample volume on the SPR assay where 50 μL of serum in 260 μL of buffer gave the maximal response (see Fig. 2). The mix was allowed to incubate for 1 h in a water bath at 37 °C. The solution was then centrifuged at 3000 ***g*** for 10 min at 4 °C and supernatant discarded. The precipitate was washed by re‐suspending in 1 mL of SA buffer and mixed prior to centrifugation at 3000 ***g*** for 10 min at 4 °C. This was repeated twice and each time supernatant discarded. The final precipitate was re‐suspended in 200 μL of distilled water and vortex mixed until proteins had re‐dissolved.

The identity of the protein in the precipitate was examined by two‐dimensional electrophoresis (2DE) as described (Braceland *et al*. 2013). Protein concentration was determined via Bradford assay (Sigma‐Aldrich), and 2DE of the protein pellet was carried out. In total, 36 protein spots from the resulting 2DE gel were excised and subjected to trypsin digestion before protein identification via electrospray ionization (ESI) mass spectrometry on an Amazon ion trap MS/MS (Bruker Daltonics) at Glasgow Polyomics (University of Glasgow). Protein identification was determined by gene ontology (Uniprot). A score of >40 was used as the acceptance threshold.

### Statistical analysis

To understand varying precipitation potential of samples and how this might correlate with the severity of tissue damage in order to establish any link between precipitation and pathological damage to tissues, individual OD∆_340_ were compared to individual histopathological score of each tissue via a general linear model procedure in SAS version 9.3 (SAS Institute) for regression analysis. Coefficient of variance was calculated as the ratio of the standard deviation to the mean, expressed as a percentage.

## Results

### The optimized selective precipitation reaction assay

**Figure 1:**
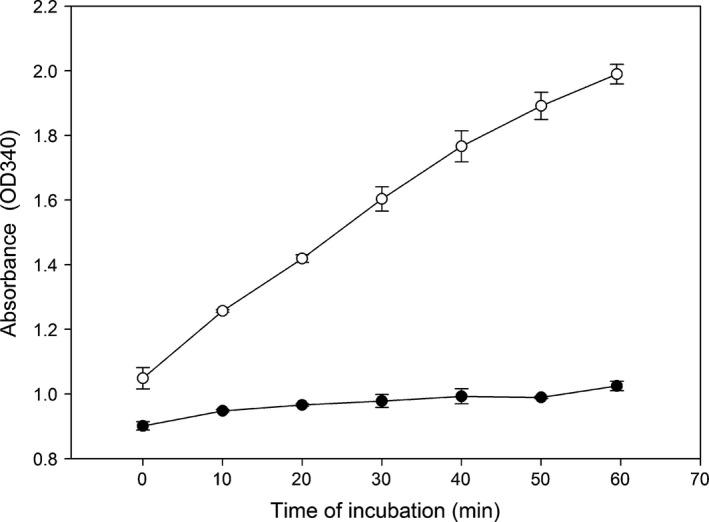
The optimized selective precipitation reaction. Optimized SPR with serum from salmon with pancreas disease at W4pc (open circle) or healthy PLA (closed circle) added to 0.6 m SA pH 5.6 and incubated at 37 °C showing mean ± SD of duplicate samples.

**Figure 2:**
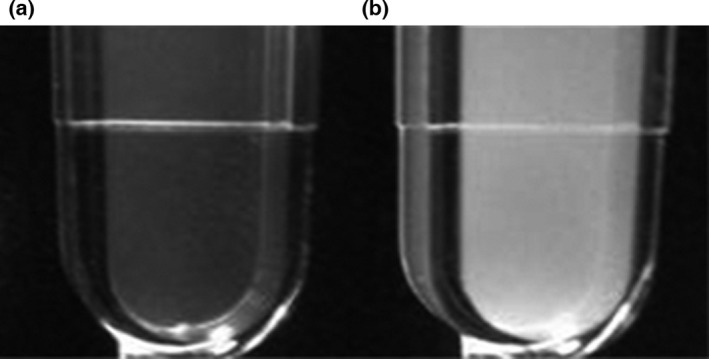
The qualitative selective precipitation reaction. Qualitative difference found in turbidity when salmon sera (15 μL) from healthy or diseased fish were mixed with 0.6 m SA buffer at pH 5.6 (245 μL) (a) PLA serum (b) W4pc serum.

A 0.6 m pH 5.6 SA buffer was found to be optimal for differentiating between healthy and diseased salmon sera. The progress of the SPR during incubation at 37 °C for 60 min is shown in Fig. 1. The mean ODΔ340 nm of pooled sera from diseased salmon (W4pc from the SAV infection trial) was higher compared to sera from the pool of healthy salmon serum (PLA) giving absorbance changes of 0.94 ODΔ340 nm and 0.12 ODΔ340 nm, respectively, after 60 min and is shown in Fig. 2 demonstrating the outcome of a qualitative SPR test. Addition of 15 μL of serum from the W4pc salmon to 245 μL of 0.6 m SA buffer at pH 5.6 produced an opaque precipitate in the assay cuvette, whereas the serum from healthy fish in the PLA gave minimal precipitation. The precipitation was stable for prolonged period (>3 days) of time at room temperature and no precipitate developed in the cuvette with the serum pool from healthy fish.

**Figure 3:**
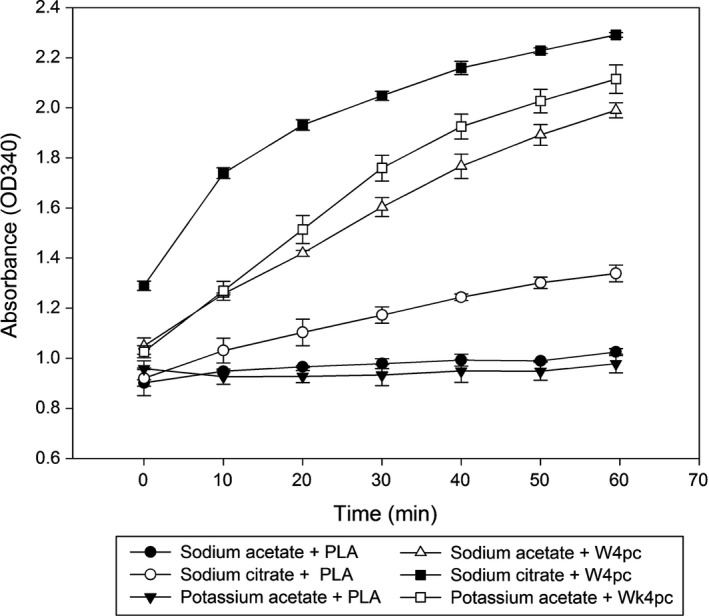
The effect of different buffers in the selective precipitation reaction. The alternative buffers, sodium citrate and potassium acetate, were used in to determine their effect on the SPR in comparison with serum from healthy salmon (PLA) to that from salmon with pancreas disease (W4pc). Results are mean ± SD of duplicates.

**Figure 4:**
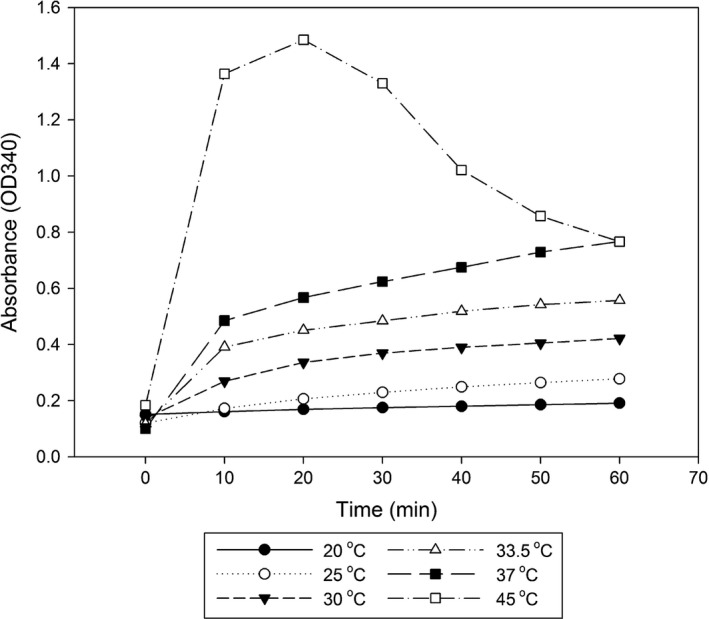
The effect of temperature on the selective precipitation reaction. Temperature effects on optical density at 340 nm over 60 min for addition of salmon muscle lysate to 0.6 m SA (1:5 v/v) where the reaction was incubated at 20, 25, 30, 33.5, 37 or 45 °C and shows the mean of three replicates in a microtitre plate.

Optimization of the SPR covered a number of assay conditions. A number of alternative buffers to SA were assessed with differential ODΔ340 nm being observed between sera from healthy salmon in PLA or diseased (W4pc) salmon using 0.6 m sodium citrate or potassium acetate buffers at pH 5.6 in the SPR assay (Fig. 3) with SA being the optimal choice. Precipitation with ammonium sulphate between 10% and 50% relative saturation (w/v) was also assessed, but this reagent precipitated serum protein from both healthy and diseased salmon with no differential precipitation between these samples. Increasing the temperature for the assay from 20 °C to 37 °C caused an increase in absorbance at 340 nm for the precipitation reaction (Fig. 4). At 45 °C absorbance increased with time initially then reduced significantly, however sedimentation of the precipitate was observed during reaction at this temperature. The effects of variation in sample volume, pH and molarity of the SA buffer and the wavelength of reading the increase in absorbance are shown in the Figures S1–S4, respectively, and were incorporated into the assay protocol. The optimized assay was validated and gave precision values of intra‐assay CV of 7.2% and interassay CV of 10.1% (*n* = 25 plates) for the SPR of sera from diseased salmon. The reference interval for SPR in serum from healthy salmon was found to be between 0.06 and 0.18 OD∆340 (*n* = 365).

### Use of SPR in a SAV infection challenge

**Figure 5:**
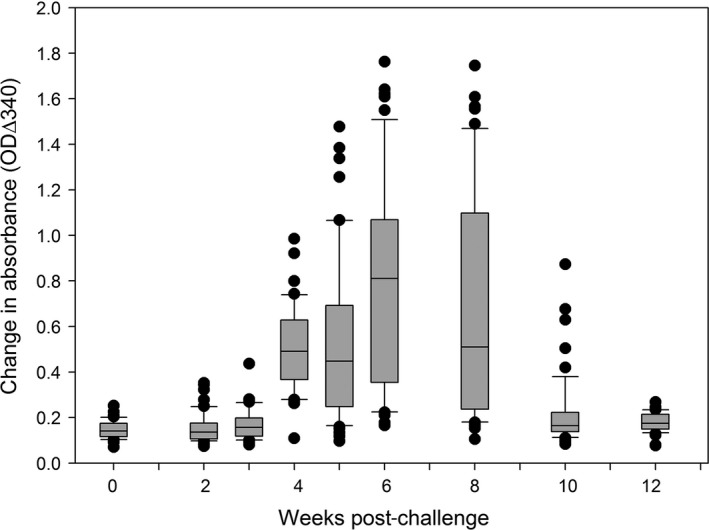
The selective precipitation reaction in an experimental model of pancreas disease in salmon. Selective precipitation reaction measured by change in absorbance over 60 min at 340 nm (∆_340_) against week post‐challenge of salmon (*n* = 54 at each time point) with salmonid α‐virus, the causative virus of pancreas disease. The box and whisker plots for each time point show the median, 10th, 25th, 75th and 90th percentiles as well as outliers.

Using the quantitative SPR assay, mean OD Δ340 increased in sera samples post‐W3pc, reaching peak ODΔ340 at W6pc and returning to basal level at W12pc (Fig. 5). The mean OD Δ340 of a clinically healthy pool (PLA) was comparable to that of fish at W0 prior to infection.

**Figure 6:**
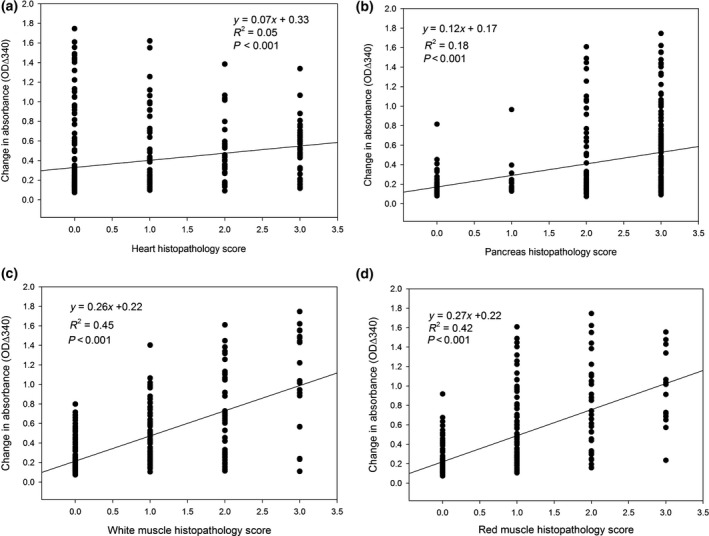
Regression analysis of the selective precipitation reaction versus histopathology score for (a) heart; (b) pancreas; (c) white muscle; (d) red muscle of salmon. Selective precipitation reaction measured by change in absorbance over 60 minutes at 340nm (OD∆_340_) in salmon during the experimental model of pancreas disease including all sampling time points and compared to histopathology score.

The statistical correlation analysis carried out to compare individual fish OD ∆340 levels against corresponding lesion scores (Table 1) showed that the SPR correlated positively with heart (Fig. 6a, *R* ^2^ = 0.05, *P* = <0.001), pancreas (Fig. 6b, *R* ^2^ = 0.18, *P* = <0.001), red muscle (Fig. 6b, *R* ^2^ = 0.45, *P* = <0.001) and white muscle (Fig. 6d, *R* ^2^ = 0.42, *P* = <0.001) histopathology scores, indicating that pathological damage to these tissues associated with the precipitate potential of serum using the SPR assay with proteins that are leaked from tissues during pathology being precipitated.

**Table 1:** Mean histopathological lesion score according to week post‐trial (Wpc)

Week Post‐challenge (WXpc)	Mean Lesion Score			
	Pancreas	Heart	Red Muscle	White Muscle
W0pc	0	0.03	0	0
W2pc	0.81	0.06	0	0
W3pc	2	1.08	0.14	0
W4pc	2.81	2.28	0.97	0.28
W5pc	2.53	1.72	1.31	0.97
W6pc	2.58	1.03	1.44	1.5
W8pc	2.39	0.11	1.31	1.89
W10pc	1.78	0.03	0.39	1.14
W12pc	1.22	0	0.03	0.08

### Proteomic investigation of the SPR precipitate

**Figure 7:**
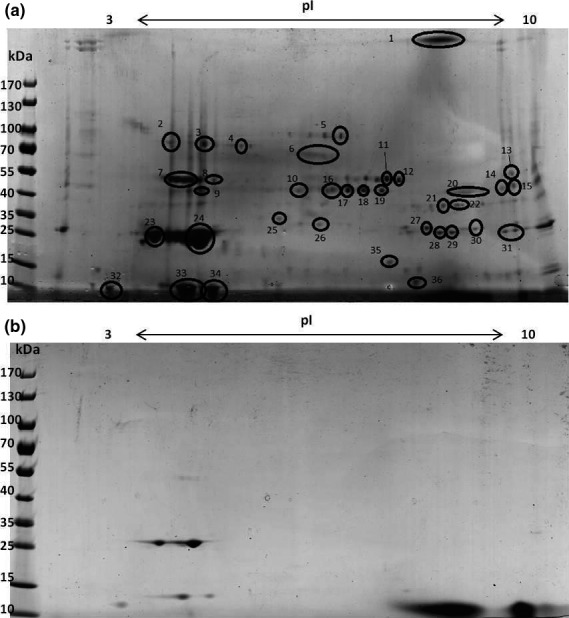
2‐DE separation of precipitate from serum of salmon with pancreas disease at W4pc (a) and serum from control fish at W0pc. The 2‐DE separation of protein content of precipitate; proteins were separated over a pI range 3‐10 and then by mass. Spots which were excised for identification are circled and numbered. W4pc serum (b) W0 serum.

Analysis of the 2DE gel profiles of precipitate from serum of SAV infected (Fig. 7a) and clinically healthy fish (Fig. 7b) revealed differential protein expression. Thirty‐six protein spots were removed for identification with mass spectrometry (File S1). The main difference in the proteome associated with the precipitation in sera from diseased fish was an increase in proteins (spots 6–22, 27–31, 35) identified as being intracellular enzymes. A number of intracellular enzymes were identified being three isoforms of creatine kinase (spots 9, 15–19, 27 and 35), enolase (spots 6–7 and 11–13), aldolase (spots 14 and 20), glyceraldehyde 3‐phosphate dehydrogenase (spots 21 and 22) and pyruvate kinase (spot 10). SPR precipitation from SAV infected salmon sera also showed increases in the serum proteins apolipoprotein A1 (spot 23–25, 33, 34), complement C9 (spots 2–4), serotransferrin (spot 5, 25), cathepsin (spot 8) and haemoglobin (spot 36). The precipitate formed with healthy fish sera was comprised of apolipoprotein A1 and haemoglobin (Fig. 7b) based on pI and molecular weight of visible protein spots.

## Discussion

This study has identified a novel precipitation reaction which occurs when serum from Atlantic salmon with PD caused by SAV infection is mixed with a sodium acetate buffer at an optimized molarity of 0.6 m and at pH5.6. In the presence of tissue damage in the salmon caused by the infection, a visible and measurable precipitate develops which is not apparent when serum from healthy fish is treated in the same way. The discovery occurred during an investigation into the acute phase protein (Cray, Zaias & Altman 2009; Ceciliani *et al*. 2012) response during PD infection. This SPR is likely to be the result of tissue pathology caused by the SAV with proteins that leak from tissue due to pathological damage becoming unstable in the buffer, aggregating and precipitating. The reaction can form the basis of a diagnostic test on serum that could provide valuable information on the health status of salmon. Optimization has allowed the development of the SPR diagnostic assay as both a laboratory (quantitative) and on‐site (qualitative) assay. A reference interval of the SPR test was determined as being from 0.06 to 0.18 OD∆340, which will be useful for the identification of samples from salmon with PD. While the reference interval was determined on salmon of average weight 3.5 kg, the range was similar for the salmon in the prechallenge group (wk0pc) of the experimental SAV infection which were of a smaller size, weighing only 30 g at the start of the experiment. This indicates that the reference interval is valid across weights of salmon, although this will need further validation in future studies, as will assessment of affect on SPR of temperature, salinity of the water and nutritional variation. The lower variation in SPR in samples from healthy salmon, which had a threefold difference between lower and upper limit of the reference interval, was less than that of creatine kinase which was reported (Braceland *et al*. 2016) as having a 10‐fold difference from lower to higher limit (2258–20567 IU L^−1^). The lower variation of the SPR is an advantage in being able to differentiate samples from healthy and diseased fish.

The mechanism of the SPR is based on protein precipitation which, caused by ionic reagents, is an established biochemical phenomenon (Asenjo 1990). Salts such as ammonium sulphate are frequently used to precipitate and separate protein (Heide, Haupt, & Schwick 1977). Despite this well‐known effect, the use of salts as selective precipitation solutions has not previously been associated with the assessment of health in fish. Ammonium sulphate was not a suitable reagent for the SPR as it was found to cause salting out of protein in both healthy and diseased salmon sera. The SPR was not buffer dependent as it was observed that potassium acetate and sodium citrate were similarly effective which may indicate that organic ions such as acetate or citrate as a buffering anion is preferred. Sodium acetate was selected as the most suitable candidate buffer due to is ability to differentiate between healthy and diseased individuals and had a lower background reading at 0 min of incubation than when sodium citrate was used. The optimized SPR assay, using a 0.6 m SA buffer at a pH of 5.6, was thus able to differentiate sera of healthy or diseased salmon on the basis of the precipitation in either a quantitative or qualitative format. Sodium acetate buffers between pH 5.2 and pH 5.6 and from 0.2 to 1.0 m were also effective in causing the SPR.

The use of serum as a non‐destructive sample to assess health holds many advantages over current molecular, histology and antibody‐based tests (Braceland *et al*. 2015). However, due to the large individual variation seen in blood biochemistry markers such as creatine kinase, the use has so far been limited (Braceland *et al*. 2016). Serum total protein has been demonstrated to be a potential marker of health and welfare in farmed fish (Coeurdacier *et al*. 2011) but as shown by the two‐dimensional electrophoresis, the SPR is detecting a subset of proteins in the serum, with muscle derived proteins predominating.

As a diagnostic test, the SPR can be either on‐farm as a qualitative test or used in a laboratory for a quantitative assay, and in either usage provides valuable diagnostic information. The qualitative test will need further assessment of its value to aquaculture but could be a rapid onsite and inexpensive point‐of‐care qualitative test. This is likely to be extremely useful in aquaculture stock health monitoring. Production sites can often be in remote locations; therefore, a point‐of‐care test offers many advantages. The SPR test can be used in conjunction with current diagnostic tests to inform when clinical manifestations of a disease have occurred and the severity of the pathology.

Use of the quantitative assay to monitor samples from experimental SAV3 infection demonstrated that the differential SPR correlated with pathogenesis, with histology lesion scores from the pancreas, heart, red and white skeletal muscle all significantly correlating with OD∆340 for individual serum samples. The observation that the correlation was greater for white and red muscle than for heart and pancreas histopathology indicates that the precipitate formation is more dependent on muscle, either via a specific mechanism or due to the larger body mass of this tissue. As general pathology peaked and fell near end of the trial, the OD ∆340 showed a similar profile of change. The assay may not be restricted as a diagnostic test for SAV as numerous bacterial and viral diseases may cause similar pathologies thus expanding the potential application of the assay in other diseases of fish. Future proteomic studies of sera obtained along the time course may identify how the nature and source of the protein in the precipitate varied during the disease and whether this could be related to individual tissues affected. These studies could use electrophoretic separation of precipitated proteins in order to link the protein electrophoretogram to relevant tissue pathologies.

In this study, pathological damage to the tissues was the main driving force of the SPR with intracellular protein entering the circulation. It is widely recognized that during an infection with salmonid alphavirus (SAV) of which there are multiple subtypes, PD develops (Munro *et al*. 1984; McVicar 1987; McLoughlin & Graham 2007). Pancreas disease is systemic and manifests in most cases with acute necrosis of acinar cells, cardiomyopathy, and extensive necrosis and fibrosis of the skeletal muscle (McLoughlin *et al*. 2006). In addition, it has previously shown by proteomic methodologies that the serum composition during infection is heavily influenced by PD‐associated pathology (Braceland *et al*. 2013, 2015). It was demonstrated that protein in the muscle lysate is denatured and precipitated by the SA buffer, which validated the use of the muscle lysate for method optimization. Whether the blood proteins in the SPR precipitate are present due to a specific, disease‐related interaction with the buffer or due to an interaction with the myocyte proteins is not clear. However, if serum proteins co‐precipitate in a non‐specific protein effect, it would be expected that many high abundance proteins other than apolipoprotein would be present.

Infection with SAV has been the initial focus of the SPR in aquaculture. It is important to determine if the assay can be of prognostic use in other economically important viral diseases in Atlantic salmon, such as heart and skeletal muscle inflammation (HSMI), infectious salmon anaemia (ISA), infectious pancreatic necrosis (IPN) or cardio‐myopathy syndrome (CMS). With the potential of the SPR as an assay to assess general health, it should also be evaluated in other key cultured fish species. Due to genotypic differences, it is anticipated that any evaluation of the SPR in other fish species will have to be optimized although it could be that the effects of SAV on the muscle of rainbow trout could lead to a similar serum reaction (Biacchesi *et al*. 2016). As an indicator of muscle damage, a potential future application of the assay may be to evaluate flesh quality prior to harvest. Viral myopathies such as SAV are widely acknowledged to cause flesh quality issues, such as bleaching of pigment and melanization (Taksdal *et al*. 2012). An increase in SPR prior to slaughter could act as an indicator of poor product quality, enabling mitigating measures to be taken to delay the harvest until the tissue pathology has resolved reducing the likelihood of downgrades at secondary processing.

The ability to make informed decisions in health management of farmed fish is of vital importance in aquaculture. Use of the SPR assay in routine monitoring of farmed salmon by non‐destructive sampling of blood may provide a rapid indication of SAV infection and associated myopathology. The qualitative SPR test may provide a rapid on‐farm assessment of health status, while the quantitative assay may provide more detailed information to a fish health professional. It is clear that the SPR assay addresses the sensitivity and specificity issues associated with the current clinical biochemistry techniques available to health professional today (Braceland *et al*. 2016). The study has demonstrated that the assay is rapid, sensitive and practical for daily operations. Moreover, the stable nature of precipitation level after the initial reaction time means that vials may be transported and investigated further using biochemical or proteomic techniques or simply to demonstrate the evidence of an issue at a site. Following a PD outbreak, use of the test along a time course may provide valuable information on the severity and/or when the pathology has resolved, thereby permitting mitigation measures to be lifted and normal farming procedures to resume for optimal management of farmed fish welfare. This study has identified and validated a novel health assessment tool, termed the SPR, which professionals can use to monitor the health of farmed Atlantic salmon.

## Authors contributions

MB carried out the SPR tests on salmon samples, the 2DE for proteomics and other laboratory investigations and co‐drafted the manuscript. JT, DC and RB provided insight into aquaculture on the SPR test, appropriate salmon serum samples and co‐drafted the manuscript. MM performed the histological analysis. PDE supervised laboratory investigations and co‐drafted the manuscript. All authors read and approved the final manuscript.

## Competing interests

JT and RB are employed by BioMar Ltd, DC is employed by Marine Harvest Scotland Ltd, and MM is employed by the Fish Vet Group Ltd. The SPR is the subject of a patent application (European Patent Application No. 14199554.8) with MB, JT, DC, RB and PDE as co‐inventors. There are no other competing interests.

## Supporting information


**File S1.** Proteins identified in the precipitate of the SPR reaction.
**Figure S1.** The effect of sample volume on the selective precipitation reaction.
**Figure S2.** The effect of pH on the selective precipitation reaction.
**Figure S3**. The effect of molarity of the buffer on the quantitative and qualitative selective precipitation reaction.
**Figure S4.** The effect of wavelength on measuring the selective precipitation reaction.
