# Supplementary material for: Selective precipitation reaction: a novel diagnostic test for tissue pathology in Atlantic salmon, Salmo salar, infected with salmonid alphavirus (SAV3)
Source: J Fish Dis. 2016 Nov 30;40(8):1077–87. doi: 10.1111/jfd.12587 (PMC5516131; doi:10.1111/jfd.12587)
Supplement: Supplementary file 1 — File S1. Proteins identified in the precipitate of the SPR reaction. Figure S1. The effect of sample volume on the selective precipitation reaction. Figure S2. The effect of pH on the selective precipitation reaction. Figure S3. The effect of molarity of the buffer on the quantitative and qualitative selective precipitation reaction. Figure S4. The effect of wavelength on measuring the selective precipitation reaction. [file JFD-40-1077-s001.doc]

**Supplementary file 1**

**Proteins identified in the precipitate of the SPR reaction.** Identities of the protein spots of the 2DE gel (fig 10) by mass spectrometry showing score, peptide matches and percent (%) sequence coverage.

| **Spot #** | **ID** | **Score** | **Matches** | **% Seq. Coverage** |
| --- | --- | --- | --- | --- |
| **1** | **No significant Matches** | **-** | **-** | **-** |
| **2** | **Complement component C9 (Q4QZ25)** | **185** | **13** | **13** |
| **3** | **Complement Component C9 (Q4QZ25)** | **408** | **32** | **19** |
| **4** | **Complement Component C9 (Q4QZ25)** | **91** | **4** | **5** |
| **5** | **Serotransferrin-1 (P80426)** | **797** | **58** | **33** |
| **6** | **Enolase 3-1 (B5DGQ6)** | **112** | **7** | **14** |
| **7** | **Enolase 3-2 (B5DGQ7)** | **261** | **24** | **26** |
|  | **Enolase 3-1 (B5DGQ6)** | **213** | **20** | **22** |
|  | **Cathespin M (Q70SU8)** | **175** | **63** | **40** |
| **8** | **Cathespin M (Q70SU8)** | **150** | **29** | **30** |
| **9** | **Fructose-bisphosphate aldolase (B5X0T0)** | **309** | **20** | **25** |
|  | **Creatine Kinase-2 (B5DGP0)** | **290** | **35** | **40** |
|  | **Creatine Kinase-3 (B5DGP2)** | **257** | **34** | **36** |
| **10** | **Pyruvate Kinase (C0H8V3)** | **109** | **4** | **6** |
| **11** | **Enolase 3-2 (B5DGQ7)** | **767** | **79** | **41** |
|  | **Enolase 3-1 (B5DGQ6)** | **635** | **71** | **39** |
| **12** | **Enolase 3-1 (B5DGQ6)** | **583** | **51** | **41** |
| **13** | **Enolase 3-2 (B5DGQ7)** | **596** | **50** | **29** |
|  | **Enolase 3-1 (B5DGQ6)** | **547** | **41** | **27** |
| **14** | **Aldolase a, fructose-bisphosphate 1 (B5DGM9)** | **251** | **16** | **26** |
| **15** | **Creatine Kinase-2 (B5DGP0)** | **400** | **57** | **42** |
|  | **Creatine Kinase-3 (B5DGP2)** | **340** | **53** | **42** |
|  | **Creatine Kinase (B5DGN9)** | **263** | **45** | **31** |
| **16** | **Creatine Kinase-2 (B5DGP0)** | **462** | **63** | **44** |
|  | **Creatine Kinase-3 (B5DGP2)** | **427** | **60** | **40** |
|  | **Creatine Kinase (B5DGN9)** | **285** | **44** | **35** |
| **17** | **Creatine Kinase-2 (B5DGP0)** | **501** | **68** | **45** |
|  | **Creatine Kinase-3 (B5DGP2)** | **452** | **64** | **45** |
|  | **Creatine Kinase (B5DGN9)** | **356** | **56** | **40** |
| **18** | **Creatine Kinase-2 (B5DGP0)** | **426** | **67** | **39** |
|  | **Creatine Kinase-3 (B5DGP2)** | **365** | **60** | **35** |
|  | **Creatine Kinase (B5DGN9)** | **191** | **36** | **27** |
| **19** | **Creatine Kinase-2 (B5DGP0)** | **491** | **77** | **39** |
|  | **Creatine Kinase-3 (B5DGP2)** | **415** | **64** | **35** |
|  | **Creatine Kinase (B5DGN9)** | **181** | **44** | **27** |
| **20** | **Aldolase a, fructose-bisphosphate 1 (B5DGM9)** | **727** | **60** | **48** |
| **21** | **Glyceraldehyde 3-phosphate dehydrogenase (O42259)** | **210** | **29** | **36** |
| **22** | **Glyceraldehyde 3-phosphate dehydrogenase (O42259)** | **251** | **31** | **48** |
| **23** | **Apolipoprotein A-I (P27007)** | **773** | **129** | **55** |
|  | **Apolipoprotein A-I-2 (O57524)** | **264** | **55** | **23** |
| **24** | **Apolipoprotein A-I (P27007)** | **1970** | **241** | **76** |
|  | **Apolipoprotein A-I-2 (O57524)** | **350** | **66** | **30** |
| **25** | **Apolipoprotein A-I (P27007)** | **571** | **92** | **56** |
|  | **Serotransferrin-2 (P80429)** | **182** | **9** | **10** |
| **26** | **Apolipoprotein A-I (P27007)** | **216** | **37** | **56** |
| **27** | **Creatine Kinase-2 (B5DGP0)** | **279** | **57** | **30** |
|  | **Creatine Kinase (B5DGN9)** | **149** | **45** | **17** |
| **28** | **Triosephosphate isomerise B (NP_001133174.1)** | **652** | **60** | **40** |
|  | **Triosephosphate isomerase (B5DGL3)** | **365** | **18** | **8** |
| **29** | **Triosephosphate isomerise B (NP_001133174.1)** | **471** | **33** | **40** |
|  | **Triosephosphate isomerase (B5DGL3)** | **295** | **14** | **8** |
| **30** | **Fructose-bisphosphate 1 (B5DGM9)** | **349** | **30** | **30** |
| **30** | **fructose-bisphosphate 2 (B5X0T0)** | **332** | **26** | **28** |
| **31** | **Triosephosphate isomerase (Q70I40 )** | **197** | **9** | **27** |
| **32** | **Type-4 ice-structuring protein LS-12** | **664** | **33** | **54** |
| **33** | **Apolipoprotein A-I (P27007)** | **149** | **24** | **52** |
|  | **Apolipoprotein A-I-2 (O57524)** | **131** | **12** | **23** |
| **34** | **Apolipoprotein A-I (P27007)** | **214** | **52** | **39** |
|  | **Apolipoprotein A-I-2 (O57524)** | **154** | **16** | **46** |
| **35** | **Creatine Kinase-3 (B5DGP0)** | **112** | **13** | **19** |
| **36** | **Hemoglobin subunit beta (Q91473)** | **385** | **39** | **79** |
|  | **Alpha-globin 1 (A9YVA1)** | **90** | **5** | **18** |

**Figure S1**

**
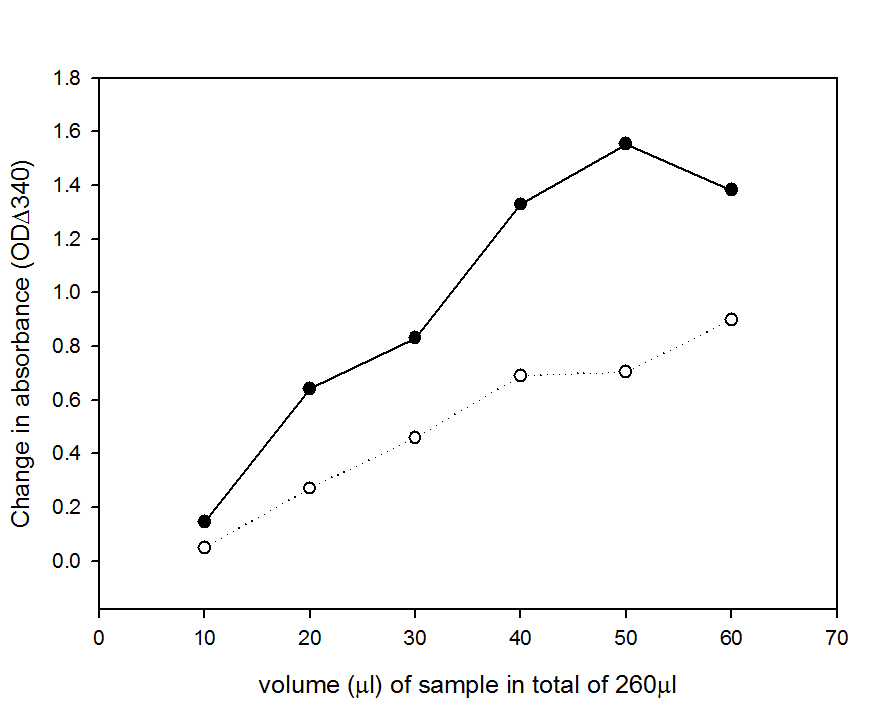
**

**Figure S1** **The effect of sample volume on the selective precipitation reaction.**

Increasing volumes of serum from salmon with pancreas disease (W4pc) and effect on the serum precipitation reaction, when added to the optimised reagent keeping the final volume at 260 ml. The graph is the mean of three replicates in a microtitre plate.

**Figure S2**

**
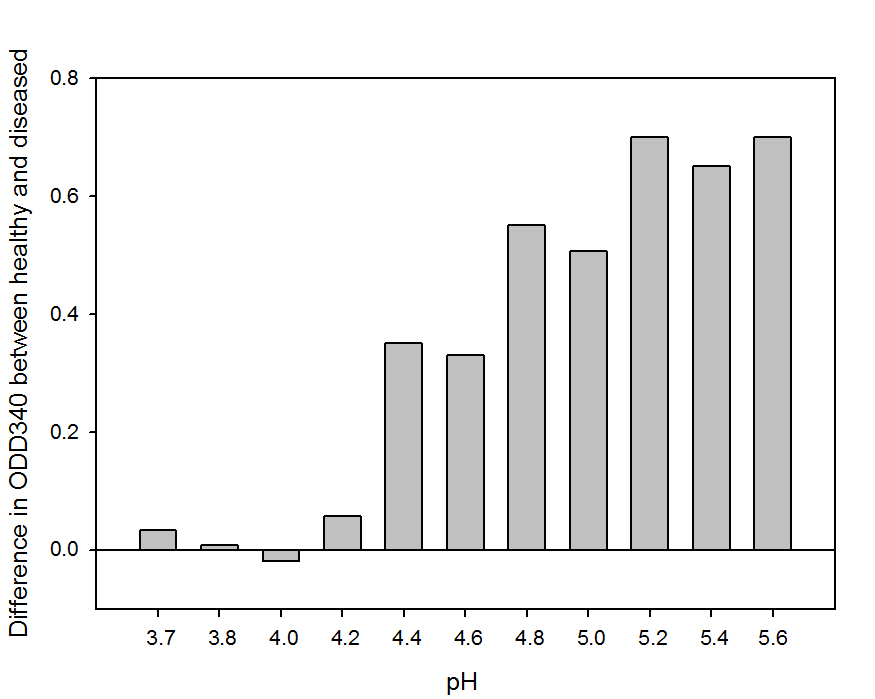
**

**Figure S2: The effect of pH on the selective precipitation reaction.**

Absorbance difference between healthy and diseased salmon (∆340 diseased - ∆340 healthy) when serum pools are introduced to 0.6M SA buffer at various pH, and shows the mean of three replicates in a microtitre plate.

**Figure S3**

**
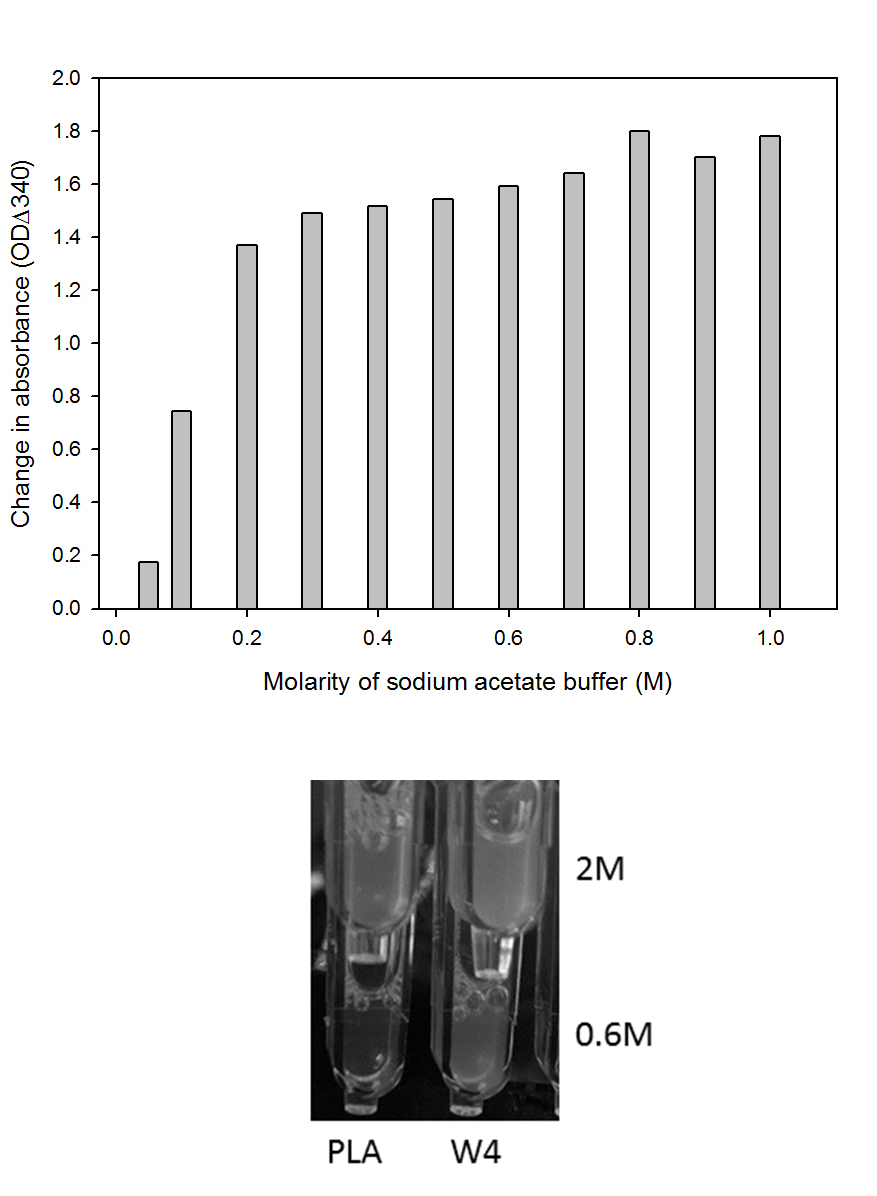
**

**A**

**B**

**Figure S3 The effect of molarity of the buffer on the quantitative and qualitative selective precipitation reaction.**

S3A: The effect of increasing the molarity of sodium acetate on the change in absorbance over 60 min in the quantitative SPR and is the mean of three replicates in a microtitre plate.

S3B: The visual turbidity when healthy (PLA) or PD positive (Wpc4) salmon sera is added to sodium acetate buffer at pH 5.6 at 0.6M or 2M ionic strength and is the mean of three replicates in a microtitre plate .

**
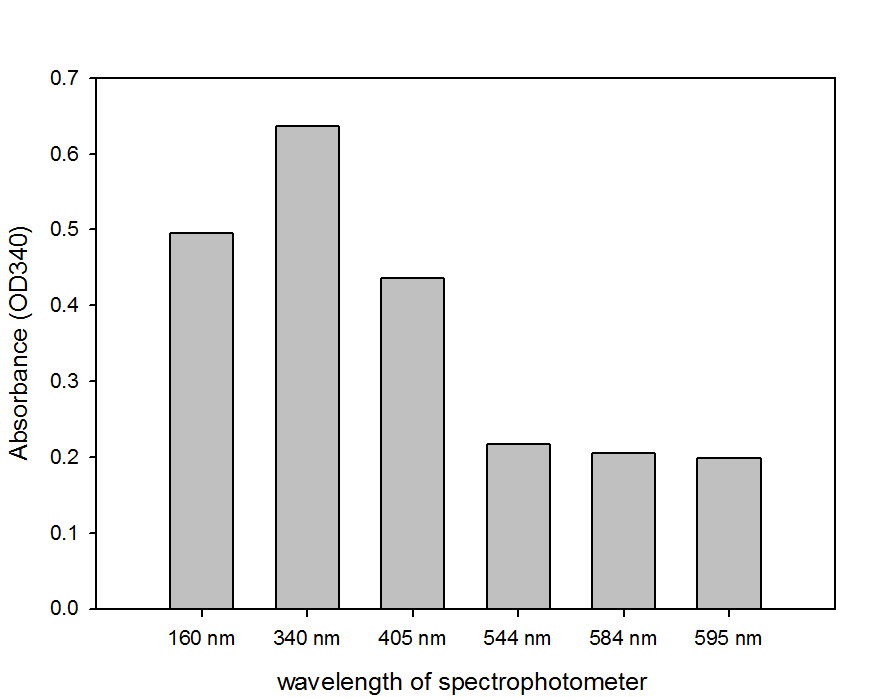
**

**Figure S4: The effect of wavelength on measuring the selective precipitation reaction.**

Optical density readings at differing wavelengths of the SPR reaction (muscle lysate in SA buffer) compared to buffer blank after 60 min incubation at 37°C and is the mean of three replicates in a microtitre plate.
